# The chronological sequence of somatic mutations in early gastric carcinogenesis inferred from multiregion sequencing of gastric adenomas

**DOI:** 10.18632/oncotarget.9250

**Published:** 2016-05-09

**Authors:** Chul-Hyun Lim, Yu Kyung Cho, Sang Woo Kim, Myung-Gyu Choi, Je-Keun Rhee, Yeun-Jun Chung, Sug-Hyung Lee, Tae-Min Kim

**Affiliations:** ^1^ Division of Gastroenterology, Department of Internal Medicine, Seoul St. Mary's Hospital, College of Medicine, The Catholic University of Korea, Seoul 137-701, South Korea; ^2^ Department of Medical Informatics, College of Medicine, The Catholic University of Korea, Seoul 137-701, South Korea; ^3^ Cancer Evolution Research Center, College of Medicine, The Catholic University of Korea, Seoul 137-701, South Korea; ^4^ Integrated Research Center for Genome Polymorphism, College of Medicine, The Catholic University of Korea, Seoul 137-701, South Korea; ^5^ Department of Microbiology, College of Medicine, The Catholic University of Korea, Seoul 137-701, South Korea; ^6^ Department of Pathology, College of Medicine, The Catholic University of Korea, Seoul 137-701, South Korea; ^7^ Cancer Research Institute, College of Medicine, The Catholic University of Korea, Seoul 137-701, South Korea

**Keywords:** gastric adenoma, gastric dysplasia, exome sequencing, mutation, multiregion sequencing

## Abstract

Mutation profiles and intratumoral heterogeneity are not well understood for benign gastric adenomas, some of which progress into malignant gastric adenocarcinomas. In this study, we performed whole-exome sequencing of three microsatellite stable (MSS) and two microsatellite instability-high (MSI-H) gastric adenomas with three regional tumor biopsies per case. We observed that the mutation abundance of benign gastric adenomas was comparable to those of gastric adenocarcinomas, suggesting that the mutational makeup for gastric carcinogenesis may already be achieved in benign adenomas. The extent of intratumoral heterogeneity was more substantial for MSS genomes in that only 1% - 14% of somatic mutations were common across the regional biopsies or ‘public’, while 50% - 94% of mutations were public in MSI-H gastric adenomas. We observed biallelic, loss-of-functional events of *APC* with truncating mutations and/or 5q losses for all cases, mostly as public events. All MSS gastric adenomas also harbored *ARID2* truncating mutations, often as multiple, region-specific ones indicative of convergent evolution. Hotspot missense mutations on known cancer genes such as *ERBB2* and *KRAS* were largely observed as region-specific aberrations. These findings suggest that biallelic functional *APC* inactivation initiates the gastric carcinogenesis and is followed by mutations of histone modifiers and then activation of known cancer-related genes. As the first exome-wide multi-region mutational profiling of gastric adenomas, our study provides clues on the chronological sequence of somatic mutations and their clonal architectures in early gastric carcinogenesis.

## INTRODUCTION

Gastric adenomas, localized polypoid proliferation of dysplastic epithelium of the stomach, are the most common type of gastric neoplastic polyps [1]. Frequently occurring in the background of chronic atrophic gastritis with mucosal atrophy [2], gastric adenomas are considered neoplastic lesions with malignant potential, and endoscopic resection is the standard management. Chronic inflammation in the gastric mucosa develops into atrophy, intestinal metaplasia, and eventually to intestinal type gastric adenocarcinoma [3]. The key molecular events that occur during early malignant transformation may be recorded in the mutation profiles of gastric adenomas. Accordingly, the investigation of genomic profiles of gastric adenomas may advance our understanding of molecular mechanisms underlying early steps in the malignant transformation of normal gastric mucosa and facilitate the screening of early acquired genomic alterations as appropriate targets for targeted therapeutics [4].

It is widely recognized that gastric cancers represent a heterogeneous set of genomic disorders and that such heterogeneity may come from variability in the genetic background of patients (i.e., inter-individual germline and somatic mutational heterogeneity) as well as intratumoral heterogeneity (ITH) [5]. The wide-spread prevalence and clinical importance of ITH has been recently recognized across various tumor types and may add a level of complexity to designing personalized treatment regimens [6–9]. For example, the regional biases of *ERBB2* amplification in gastric cancers may reduce the efficacy of targeted treatment [10]. Mutational ITH may serve as the source for the development of treatment resistance, and a recent study reported that a higher level of ITH may be associated with unfavorable clinical outcome in gastric cancers [11]. The investigation of ITH for gastric adenomas as premalignant lesions may provide clues about when and how ITH arises during gastric carcinogenesis.

In this study, we performed whole-exome sequencing of five gastric adenomas — three microsatellite stable (MSS) and two microsatellite instability-high cases (MSI-H) — each of which was profiled for three regional biopsies and matched normal genomes. The sequencing data was used to identify somatic mutations of single base substitutions or SNV, short insertions/deletions or indels and microsatellite instability (MSI), as well as DNA copy number profiling. Our strategy of multiregion sequencing and mutation profiling provided a means to not only measure the level of ITH, but also categorize the mutations according to regional distribution (e.g., those present across all regional biopsies or region-specific mutations as public and private, respectively). Given that such spatial mutation categories largely correspond to the temporal order of their occurrence during the evolution of cancer, the chronological sequence of somatic mutations was inferred for early gastric carcinogenesis.

## RESULTS

### Mutational abundance in gastric adenomas

**Figure 1:**
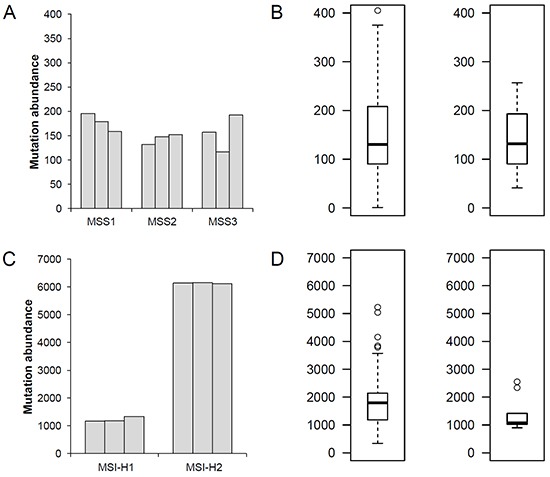
Mutation abundance of MSS and MSI-H gastric adenomas **A.** For three MSS gastric adenomas (MSS1-MSS3), the number of exonic mutations (mutation abundance) are shown for three regional biopsies per case. **B.** The number of exonic mutations is shown for the eight MSS gastric adenocarcinomas analyzed by the same sequencing platforms and analysis pipeline for this study (Left) and 218 MSS gastric adenocarcinomas from the TCGA consortium (Right). **C.** Exonic mutation abundance of two MSI-H gastric adenomas (MSI-H1 and MSI-H2). **D.** Mutation abundance of 9 and 70 MSI-H gastric adenocarcinomas from the previous study and the TCGA consortiums, respectively, are shown.

The clinicopathologic information of three MSS (MSS1-3) and two MSI-H (MSI-H1-2) gastric adenoma patients is shown in Table 1. We first investigated three types of somatic mutations — SNV, indels and MSI — across three regional biopsies per case (see Methods and Materials). We only considered genomic alterations on coding sequences (i.e., exonic mutations). First, we measured the coding mutation abundance for the five gastric adenoma cases separately for MSS and MSI-H cases (Figure 1). For comparison, the mutational abundance of gastric adenocarcinomas was obtained from our previous study where 8 MSS and 9 MSI-H gastric adenocarcinomas were analyzed using the same sequencing platforms and the analysis pipeline of this study [12] and also from a large public database of the Cancer Genome Atlas (TCGA) consortium with 218 MSS and 70 MSI-H gastric adenocarcinomas [13]. The mutational abundance of MSS gastric adenomas (120 - 180 mutations per genome; median of 157 mutations) is largely comparable to that observed for MSS gastric adenocarcinomas in our study and the TCGA consortium (median of 132 and 130 mutations per case, respectively, Figure 1A-1B). This similarity was also true for MSI-H gastric adenomas, one of which (MSI-H2) showed an extremely high mutation rate (>6100 exonic mutations corresponding to 120 mutations per Mb) (Figure 1C-1D). This finding suggests that the mutational makeup for malignant transformation develops early in the benign stages during gastric carcinogenesis.

**Table 1:** Clinicopathologic information of five gastric adenoma patients

Case	Age	Gender	Location	Stage	Pathology	Size (cm)	*Helicobacter Pylori*
MSS1	75	M	Antrum	IIb	Low grade dysplasia	2.6 × 1.7	+
MSS2	72	M	Body	IIb	Low grade dysplasia	3.2 × 1.8	-
MSS3	70	M	Angle	IIb	High grade dysplasia	4.4 × 1.6	+
MSI-H1	71	M	Antrum	IIa	High grade dysplasia	2.4 × 1.2	-
MSI-H2	75	M	Body	IIb	High grade dysplasia	4.2 × 2.7	-

### Mutational ITH of gastric adenomas

**Figure 2:**
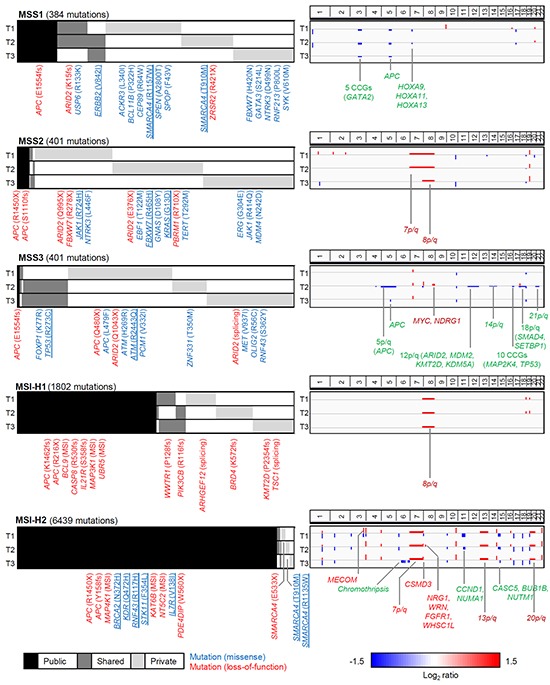
Landscape of somatic mutations and copy number alterations of gastric adenomas For each of the five gastric adenomas (MSS1-3 and MSI-H1-2; from top to bottom), somatic mutations and genome-wide copy number profiles are shown in left and right panels, respectively. Per case, three lanes (T1-3) represent the three regional tumor biopsies. For mutations, public (black), shared (dark grey) and private mutations (light grey) are shown with their regional distribution. Among the mutations occurring on known cancer-related genes, the loss-of-function events (i.e., nonsense or splicing mutations and out-of-frame indels; red) and missense mutations (blue) are annotated. The missense mutations at hotspots (>= 5 occurrences in COSMIC database; blue underbar) are also shown. In the case of MSI-H adenomas, only the missense mutations at hotspots along with loss-of-function mutations are shown. For copy number alterations, the copy number gains and losses are shown as red and blue, respectively. The known cancer genes that belong to the copy number gains and losses are shown as gene symbols in red and green, respectively. The arm-level alterations are also annotated as such. The chromothripsis-like event observed in MSI-H2 genomes is also indicated.

To consider the spatial distribution across three regional biopsies, we categorized somatic mutations according to the number of observed regional biopsies in given cases (3, 2 and 1 observed biopsies for public, shared and private mutations, respectively). To prevent the overestimation of ITH, we reevaluated somatic mutations by examining the presence of sequencing reads supporting the mutations found in other regional biopsies (see Methods and Materials and Supplementary Figure S1). Overall, mutational ITH was evident for the three MSS gastric adenomas where the public mutations comprised only 14.3%, 4,0% and 0.7% of the total mutations observed in the MSS1-MSS3 cases, respectively (black in mutation profiles; Figure 2). For the two MSI-H cases, in contrast, 50.1% and 93.7% of mutations were public, suggesting that the extent of mutational ITH of MSI-H gastric adenomas is less substantial compared to MSS cases.

### Landscape of somatic mutations and copy number alterations in gastric adenomas

The mutational landscape of gastric adenomas is shown along with genome-wide copy number profiles in Figure 2. For each of the five gastric adenomas sampled, mutations and copy number alterations of three regional biopsies are illustrated in the order of mutation recurrence (public-shared-private; mutations, Figure 2, Left) and genomic coordinates (copy number alterations; Figure 2, Right), respectively. The full list of somatic mutations for the five gastric adenomas is available in Supplementary Tables S1 to S5.

In MSS1, one *APC* truncating mutation (p.E1554fs) accompanied by focal 5q loss involving *APC* was observed. Both *APC* alterations were public events (i.e., observed in all the regional biopsies and thus, regionally clonal), suggestive of early, clonal billallelic inactivation of *APC* in this case. In addition, a truncating mutation of *ARID2* (p.K15fs) was observed as a shared event involving two out of three regional biopsies. Each of two regional biopsies harboring shared *ARID2* truncating mutations also showed private missense mutations of another chromatin remodeler, *SMARCA4,* on known hotspots (p.R1157W and p.T910M; >= 5 occurrences in COSMIC database) [14]. An *ERBB2* hotspot mutation (p.V842I) was also observed as a shared mutation. This mutation has been identified as one of seven functionally validated *ERBB2* activation mutations [15]. Region-specific *ERBB2* amplification with potential clinical relevance has been previously reported [10].

In MSS2, two truncating, public mutations of *APC* in the form of one out-of-frame indel (p.S1110fs) and one nonsense mutation (p.R1450X) were observed, also suggesting early, biallelic *APC* inactivation. In addition, two private truncating *ARID2* mutations (p.Q995X and p.E376X) were observed as regionally exclusive multi-hits, providing evidence for potential functional convergence. A truncating mutation of *PBRM1* (p.R710X) as an additional loss of a chromatin modifier, was also observed in the biopsies harboring *ARID2* mutations. The well-known *KRAS* missense mutation (p.G13D) was observed as a private event.

In MSS3, the *APC* inactivation appeared as biallelic, but in a mosaic pattern. One public, truncating mutation of *APC* (p.E1554fs) was common across all three regional biopsies, each of which harbored one private truncating *APC* mutation (p.Q480X) and region exclusive, private copy number alterations of arm-level 5q loss and focal 5q loss involving *APC*. Of interest, this functional convergence was also observed for *ARID2,* with three regionally exclusive, private inactivating events of one nonsense (p.Q480X) and one splicing mutation, as well as 12p/q loss involving *ARID2*. In addition, hotspot mutations of *TP53* (p.R273C) were noted in two regional biopsies. Further, the *ATM* hotspot mutation (p.R2443Q) appeared in the remaining, *TP53* wildtype biopsy, suggesting potential pathway-level functional convergence.

Biallelic, public *APC* inactivations were also observed for two MSI-H gastric adenomas, in that each genome contained one nonsense mutation and one out-of-frame indel involving *APC* (e.g., p.R215X/p.K1452fs for MSI-H1 and p.R1450X/p.Y158fs for MSI-H2, respectively). In the MSI-H1 genome, most truncating mutations involving cancer-related genes were observed as out-of-frame indels. These genes included chromatin modifier *BRD4* and histone methyltransferase *KMT2D* (both as private) as well as genes that belong to a various cellular functions such as *BCL9, CASP8, IL21R, MAP3K1, UBR5* (all public). Unlike MSI-H1, with a relative deficit of copy number alterations except for public 8q gains, MSI-H2 had prevailing copy number alterations along with a characteristic signature of chromothripsis (e.g., alternating copy number states of eight focal segments) in chromosome 6. Given the extremely high mutational burden of MSI-H2, the interpretation of prevailing missense mutations requires caution, but we observed potential biallelic hotspot mutations on *SMARCA4*. Due to its role as a chromatin modifier and the recurrent nature (regionally exclusive hotspot mutations in MSS1), we propose that *SMARCA4* mutations may play a role in early gastric carcinogenesis, along with *ARID2*.

### Chronological sequences of somatic mutations in gastric adenomas

**Figure 3:**
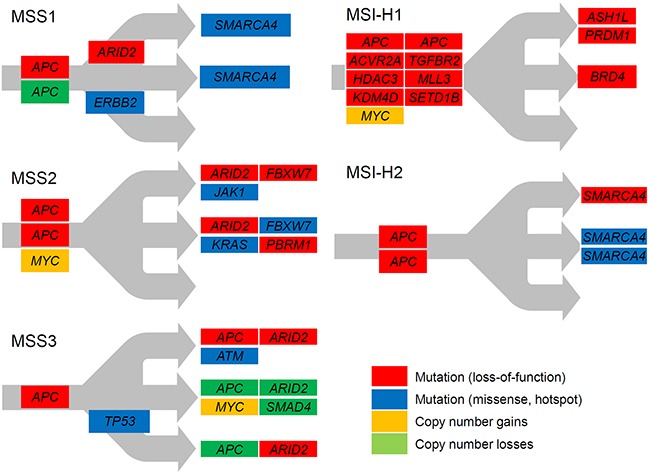
Schematics of regional evolution of gastric adenomas Mutations on cancer-related genes are shown according to the regional distribution. Mutations on the trunk (public) and branches (shared) are distinguished from private mutations. Red and blue represent the loss-of-function and hotspot missense mutations, respectively. Orange and green represent genes with copy number gains and losses, respectively.

The schematics of temporal orders in the acquisition of key somatic mutations are shown in Figure 3. It has been proposed that clonal-*vs*-subclonal mutations can be distinguished from each other based on mutant- or variant-allele frequencies from mass-level sequencing [16]. Spatial mutation categorization using multiregion sequencing is a more advanced methodology that enables the investigation of ITH as well, with the determination of temporal orders between somatic mutations [6, 9, 17]. The schematics of MSS1 and MSS2 show that these genomes had biallelic *APC* inactivation as initiating events and subsequently acquired truncating *ARID2* mutations as well as truncating or hotspot mutations on other components of SWI/SWF complexes, such as *SMARCA4* (MSS1) and *PBRM1* (MSS2). It is evitable that the mutations on known cancer genes such as *KRAS*, *FBXW7* and *ERBB2* are evolutionarily acquired later, after the biallelic *APC* inactivation, suggesting that the multiregion sequencing of gastric adenomas may illustrate the temporal order of key somatic mutations in early gastric carcinogenesis. The MSS3 genome exceptionally showed the characteristic mosaic patterns for *APC* and *ARID2,* suggesting that this genome may represent early stages of gastric carcinogenesis before clonal sweeps, and that key initiating events such as *APC* and *ARID2* inactivation may occur as multiple, functionally converging events among which the subclones with the best fitness will be selected and survive.

The two MSI-H gastric adenomas showed elevated mutations rates (23 and 123 mutations per Mb for MSI-H1 and MSI-H2, respectively). The ratio of nonsynonymous-*vs*-synonymous (NS/S) mutations of MSI-H1 (NS/S ratio = 2.0) was similar to that of the MSS gastric adenomas (NS/S ratio = 2.1 − 2.4) but the NS/S ratio of MSI-H2 was substantially lower (0.99). MSI-H1 genomes showed a substantially higher number of out-of-frame indels and MSI mutations compared to MSI-H2 genomes, with dominance of SNV. Consistent with this observation, the MSI-H1 genome displayed the inactivation of *ACVR2A* and *TGFBR2* as frequent targets of MSI mutations along with a number of epigenetic regulators also targeted by out-of-frame indels and MSI mutations. In contrast, the MSI-H2 genome showed a dominance of SNVs without characteristic MSI events on *ACVR2A* or *TGFBR2*. No *POLD1* or *POLE* mutations, previously recognized causal events for elevated SNV rates without a relative increase of indels [18], were observed for MSI-H2, indicating a need for further investigation for this unique mutator phenotype.

## DISCUSSION

In this study, we performed whole-exome sequencing-based mutation and DNA copy number profiling for five gastric adenomas as premalignant lesions of intestinal type gastric adenocarcinomas. Given that whole-genome or -exome scale mutation profiling is not yet available for gastric polyps or adenomas, our results provide, for the first time, whole-exome scale mutation profiles as well as evolutionary insights, including the temporal order of somatic mutations in early gastric carcinogenesis. For multiregion mutation profiling, each case was analyzed with three regional tumor biopsies along with matched normal genomes (15 tumor and 5 matched normal whole-exome sequencing data in total). Along with the inspection of genomic ITH, multiregion sequencing enabled us to prioritize the mutations according to regional distribution (e.g., public, shared and private). These spatial mutational categories are also assumed to be correlated with the temporal order of somatic mutations in a given cancer genome. For example, the public mutations have occurred in the founding clone that may have undergone a number of clonal sweeps during expansion, while private mutations have occurred in late evolving clones that have not been subject to clonal sweeps [7, 8, 19]. Since the advanced cancer genomes are more likely to have the chance of clonal sweeps, losing the archetypal, subclonal mutational architecture of early acquired somatic mutations, the investigation of gastric adenomas as premalignant lesion of gastric cancers may provide valuable information regarding the temporal orders of somatic mutations of cancer-initiating events.

First, public truncating mutations involving *APC*, a well-known gate-keeping gene, were observed in all of the gastric adenomas examined, suggesting that *APC* inactivation is the universal, initiating event in the development of gastric adenomas. Notably, all the gastric adenomas also showed biallelic *APC* losses with either two truncating *APC* mutations (e.g., out-of-frame indel and nonsense mutation) present or one truncating *APC* mutation present along with focal/arm-level 5p losses in the given cases. Biallelic inactivation is a hallmark of tumor suppressor genes consistent with the notion of Knudson's two-hit hypothesis [20]. Of note, the frequency of *APC* mutations in gastric adenomas in this study (100%) is comparable to those of colorectal cancers (~80% of MSS colorectal cancers), instead of those of gastric adenocarcinomas (<10%) [13, 21]. The discrepancy in the frequency of *APC* mutations between gastric adenomas and advanced adenocarcinomas raises a hypothesis that the gastric adenomas accompanying intestinal metaplasia can be initiated with *APC* inactivation mirroring colorectal carcinogenesis and comprise a relatively small fraction of fully advanced gastric adenocarcinomas. Since the primary aim of our study was to identify the regional distribution of somatic mutations in a limited number of gastric adenomas, the interpretation of the mutation frequency requires caution. Further validation in extended cohorts of gastric adenomas is needed to evaluate the hypothesis related to the frequency of somatic mutations.

Second, we observed a prevalence of somatic mutations on genes with chromatin remodeling activity, e.g., truncating mutations on *ARID2* in all three MSS gastric adenomas and also on *PBRM1* and *SMARCA4*. In the case of *ARID2*, characteristic ‘mosaic’ patterns of mutations were observed. For example, one MSS genome (MSS2) showed two biopsy-specific *ARID2* nonsense mutations and one case (MSS3) harbored three biopsy-specific *ARID2* alterations, including one nonsense and one splicing mutation, as well as a region-specific 12q loss. The occurrence of multiple somatic mutations involving the same gene across regional biopsies has been considered as evidence of functional convergence [9]. These functionally converging and regionally mosaic patterns were also observed for *APC,* where three regional biopsies of MSS3 harbored one nonsense *APC* mutation, arm-level and focal 5q losses, respectively, along with a public out-of-frame indel of *APC*. In addition, two region-specific hotspot missense mutations were observed for *SMARCA4* (MSS1) and similar representation was observed for a cancer-related gene of *FBXW7* (one nonsense and one hotspot mutation in MSS2). Potential pathway-level convergence, such as the region-specific *ATM* and *TP53* mutations, was also observed. Although previous ITH studies have reported a number of examples of converging evolution [9], the prevalence of such phenomena on key cancer genes may characterize early tumorigenesis, where multiple drivers simultaneous occur and compete with each other.

In addition, we observed that the mutation abundance of benign gastric adenomas was comparable to that of gastric adenocarcinomas for both microsatellite-stable and -unstable cases. Along with the lines of evidence suggesting that early gastric cancers show a mutation abundance comparable to advanced gastric cancers [12], this finding suggests that the acquisition of somatic mutations for gastric carcinogenesis may be achieved early during the evolution of cancer genomes. In contrast, DNA copy number alterations are relatively infrequent in gastric adenomas, except for one case in this study (MSI-H2). This finding suggests that copy number alterations in gastric adenomas may comprise later genomic events, but further investigation is needed to determine whether these later genomic events are merely the products of increased genomic instability or can confer essential survival benefits. Further, a substantial level of mutational ITH was observed for MSS gastric adenomas, which is consistent with other solid tumor types [7–9, 22]. However, the extent of mutational ITH was low in MSI-H gastric adenomas, and in one extreme case (MSI-H2), more than 90% of somatic mutations were commonly shared across all the regional biopsies examined. Although mutational ITH has been rarely studied in tumors with mutator phenotypes or elevated mutation rates, a number of hypotheses can be proposed. For example, it can be assumed that the majority of somatic mutations in hypermutable tumors may occur in a relatively short time during early cancer genome evolution and become fixed as public mutations. In addition, the increased mutation burden may prevent the acquisition of additional somatic mutations, which may explain the lack of private mutations. Alternately, the clonal sweeps that may render the majority of somatic mutations in a founding clone as public may be more frequent in MSI-H gastric adenomas and be responsible for the high proportion of public mutations in these genomes.

Finally, we observed a lack of somatic mutations previously reported as frequent mutations in gastric adenocarcinomas (e.g., *TP53* and *ARID1A*)[13, 23, 24]. Given the high prevalence of *APC* and *ARID2* mutations in other gastrointestinal tumors such as colorectal cancers and hepatocellular carcinomas [21, 25] instead of gastric adenocarcinomas, this finding suggests that gastric adenoma may represent a disease category distinguished from gastric adenocarcinomas. It is possible that gastric adenomas represent early stages of gastric carcinogenesis where late-occurring mutations such as *TP53* mutations have not yet occurred. Further investigation is needed to find out whether gastric adenoma acquires additional, essential cancer drivers such as *TP53* mutations, given that gastric adenomas have already achieved a comparable number of somatic mutations.

## MATERIALS AND METHODS

### Patients

Five patients with gastric adenomas were enrolled in the study. Approval for this study was obtained from Seoul St. Mary's Hospital institutional review board (KC14TISI0436). The lesions were resected by endoscopic submucosal dissection. Three multibiopsy tumor specimens that were at least 1cm apart were obtained during the endoscopic resection by endoscopic biopsy forceps. Patient blood was also collected in EDTA-treated tubes for a matched normal genome sample. The tissue specimens were snap-frozen and stored in liquid nitrogen. The frozen tissues were cut serially and stained with hematoxylin-eosin for histologic examination. The tumor cell purity (>70%) was confirmed by board-certified pathologists along with additional histological examination. The clinicopathologic features of the five gastric adenoma patients are summarized in Table 1.

### Whole-exome sequencing

For genomic DNA extraction, we used the DNeasy Blood & Tissue Kit (Qiagen, Germany) according to the manufacturer's recommendations. Per patient, the genomic DNA was obtained from three regional biopsies as well as from the patient blood. After quality checks, exomic DNA was captured from the genomic DNA using the Agilent SureSelect Human All Exome 50Mb kit (Agilent, USA). Genomic DNA libraries were prepared and 100bp paired-end sequencing reads were generated using the Illumina HiSeq2000 platform according to the manufacturer's recommendations (Illumina, USA). Sequencing information, including coverage, is available in Supplementary Table S6.

### Mutation profiling

To align the raw sequencing reads onto the human reference genome (hg19), we used Burrows-Wheeler aligners with the default option [26]. Local realignment and score recalibration of the sequencing reads were performed using the Genome Analysis ToolKit [27]. Additional processing and sequencing data management was accomplished with Picard and SamTools [28]. To identify somatic SNV and indel, we used MuTect and Indelocator by comparing the tumor and matched normal sequencing data, respectively [27, 29]. The ANNOVAR package was used to intersect the somatic mutations on coding sequences and annotate the functional consequences of somatic variants [30]. To compare the mutation abundance of gastric adenomas with those of gastric adenocarcinomas, we used our previous mutation call data from gastric adenocarcinomas analyzed with the same analysis protocols [12]. We also obtained large-scale mutation profile data of gastric adenocarcinomas from the TCGA consortium [13]. In evaluating ITH, we performed joint calling of somatic mutations so as not to overestimate the extent of ITH. The mutations called in any of three regional biopsies were reexamined for the presence of sequencing reads supporting the corresponding mutations. The extent of mutation ITH with the effect of joint calling of somatic mutations is shown in Supplementary Figure S1.

### MSI analyses

To examine the MSI status of regional biopsies, we performed capillary sequencing for five Bethesda markers: BAT25, BAT25, D2S123, D5S346, and D17S250. Three MSS cases with no DNA slippage events on the five examined markers were delineated from two MSI-H cases (MSI-H1 and MSI-H2) with 5 and 3 markers, respectively, showing DNA slippage events (Supplementary Figure S2). For sequencing-based MSI analysis, we used our previously proposed algorithm [31]. For 146,000 reference microsatellite repeats previously identified on coding sequences, we obtained the repeat length distribution from the tumor and matched normal genomes. Differences in the length distribution were estimated by Kolmogorov-Smirnov tests for each microsatellite repeat and the significance was adjusted for the multiple tests as previously described [31]. A significant (false discovery rates or FDR < 0.05) difference was considered an MSI event. Sequencing-based MSI calling was largely concordant with the Bethesda results in that three MSS genomes showed 0 to 5 MSI events while the MSI-H1 and MSI-H2 genomes harbored 128 and 46 MSI events, respectively.

### Copy number profiling

For copy number profiling, we used the VarScan2 algorithm to obtain read depth differences between the tumor and matched normal exome sequencing data [32]. The GC-corrected read depth was log2-transformed and segmented using circular binary segmentation algorithm [32].

## SUPPLEMENTARY FIGURES AND TABLES














