# Supplementary material for: The chronological sequence of somatic mutations in early gastric carcinogenesis inferred from multiregion sequencing of gastric adenomas
Source: Oncotarget. 2016 May 9;7(26):39758–67. doi: 10.18632/oncotarget.9250 (PMC5129968; doi:10.18632/oncotarget.9250)
Supplement: Supplementary file 1 [file oncotarget-07-39758-s001.pdf]

## SUPPLEMENTARY FIGURES AND TABLES

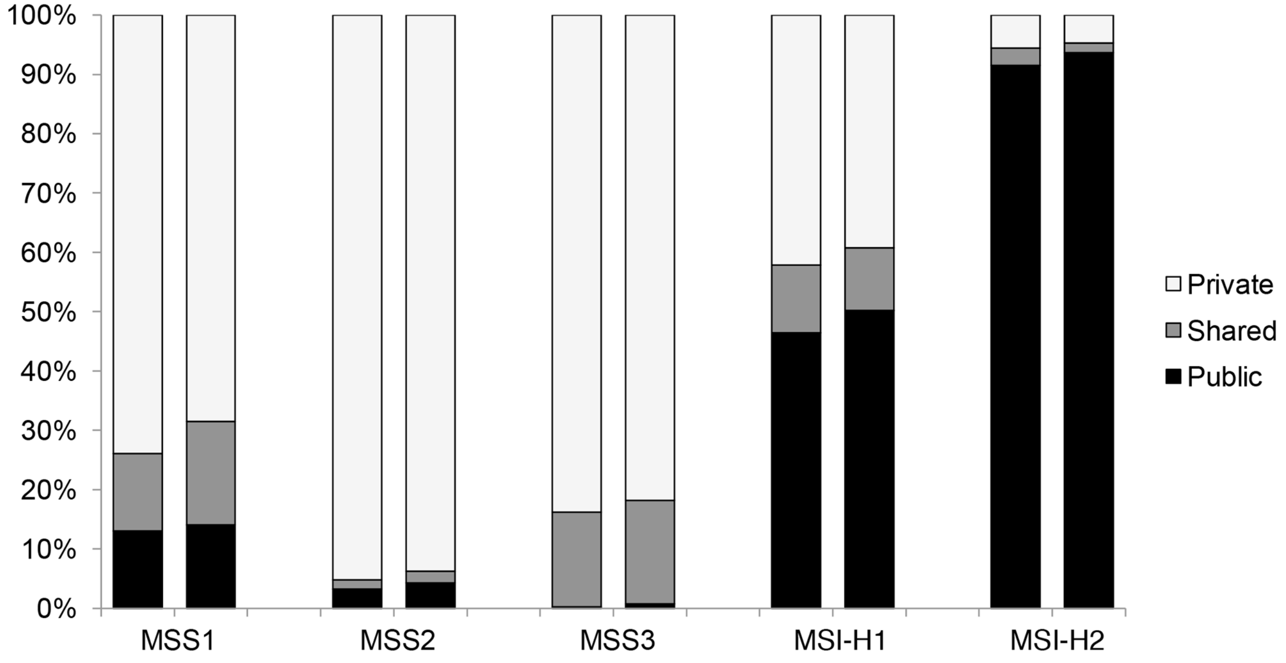


**Supplementary Figure S1: The effect of joint calling of somatic mutations.** For each of the five gastric adenomas, the relative proportion of public, shared and private mutations are shown before and after the joint calling of somatic mutations (left and right, respectively). Overall increase of public mutations were observed after the joint calling but the effect was minimal.

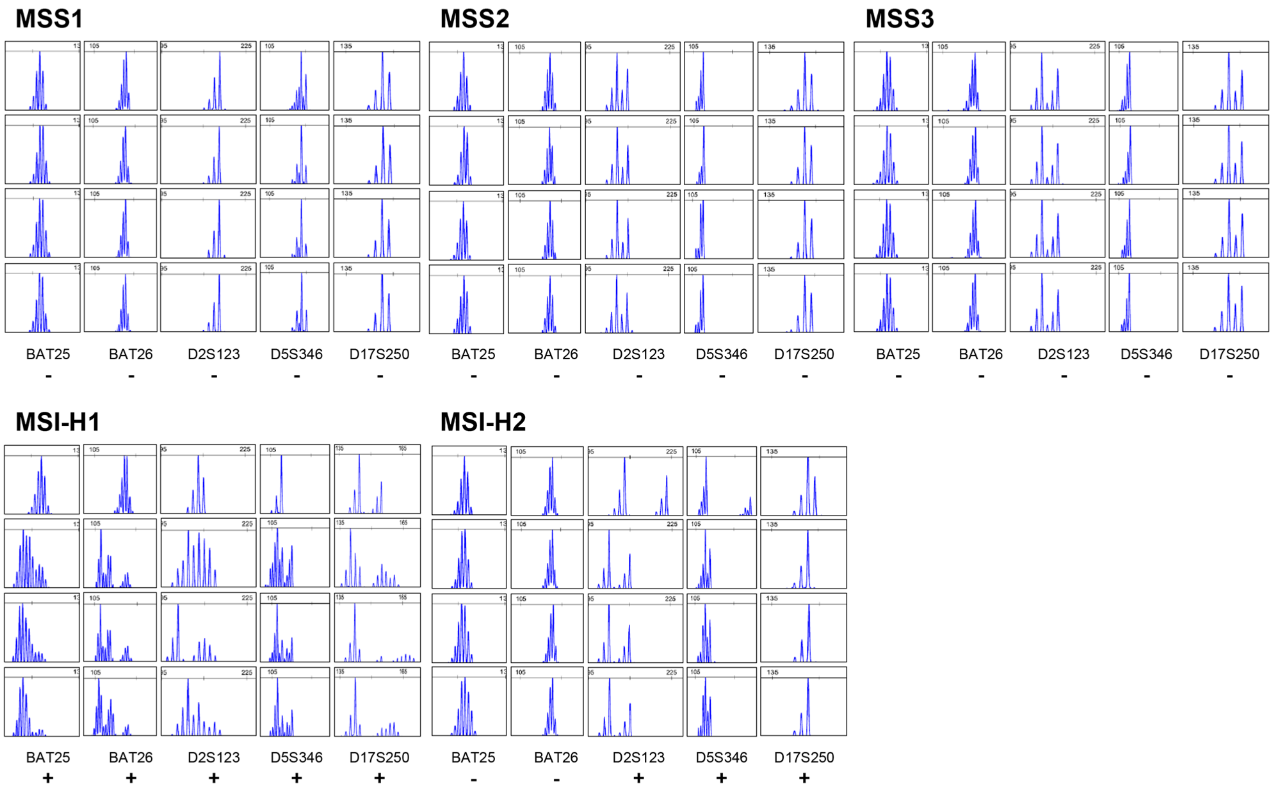


**Supplementary Figure S2: MSI status of gastric adenomas.** Five bethesda markers were used to evaluate the MSI status of gastric adenomas. Three MSS and two MSI-H genomes were distinguished according to the number of markers with apparent DNA slippage events.

**Supplementary Table S1: Somatic mutations of MSS1.** The mutation class and description are shown as annotated by ANNOVAR. Genomic coordinates (hg19) are shown. For three tumor biopsies (T1 - T3), 1 indicates the presence of the corresponding mutations.

**See Supplementary File 1**

**Supplementary Table S2: Somatic mutations of MSS2.** The mutation class and description are shown as annotated by ANNOVAR. Genomic coordinates (hg19) are shown. For three tumor biopsies (T1 - T3), 1 indicates the presence of the corresponding mutations.

**See Supplementary File 2**

**Supplementary Table S3: Somatic mutations of MSS3.** The mutation class and description are shown as annotated by ANNOVAR. Genomic coordinates (hg19) are shown. For three tumor biopsies (T1 - T3), 1 indicates the presence of the corresponding mutations.

**See Supplementary File 3**

**Supplementary Table S4: Somatic mutations of MSI-H1.** The mutation class and description are shown as annotated by ANNOVAR. Genomic coordinates (hg19) are shown. For three tumor biopsies (T1 - T3), 1 indicates the presence of the corresponding mutations.

**See Supplementary File 4**

**Supplementary Table S5: Somatic mutations of MSI-H2.** The mutation class and description are shown as annotated by ANNOVAR. Genomic coordinates (hg19) are shown. For three tumor biopsies (T1 - T3), 1 indicates the presence of the corresponding mutations.

**See Supplementary File 5**

**Supplementary Table S6: Information of whole-exome sequencing.**

**See Supplementary File 6**
